# On the Potential of a Poly(vinylidenefluoride-*co*-hexafluoropropylene) Polymer Inclusion Membrane Containing Aliquat^®^ 336 and Dibutyl Phthalate for V(V) Extraction from Sulfate Solutions

**DOI:** 10.3390/membranes12010090

**Published:** 2022-01-14

**Authors:** Salar Bahrami, Leila Dolatyari, Hassan Shayani-Jam, Mohammad Reza Yaftian, Spas D. Kolev

**Affiliations:** 1 Department of Chemistry, Faculty of Science, The University of Zanjan, Zanjan 45371-38791, Iran; bahrami_salar@znu.ac.ir (S.B.); shayan@znu.ac.ir (H.S.-J.); 2 Department of Chemistry, Zanjan Branch, Islamic Azad University, Zanjan 45156-58145, Iran; leiladolatyari@yahoo.com; 3 School of Chemistry, The University of Melbourne, Melbourne, VIC 3010, Australia; 4 Department of Chemical Engineering, The University of Melbourne, Melbourne, VIC 3010, Australia

**Keywords:** polymer inclusion membrane (PIM), poly(vinylidenefluoride-*co*-hexafluoropropylene), vanadium(V), extraction, Aliquat^®^ 336

## Abstract

A polymer inclusion membrane (PIM) composed of 50 wt% base polymer poly(vinylidenefluoride-*co*-hexafluoropropylene), 40 wt% extractant Aliquat^®^ 336, and 10 wt% dibutyl phthalate as plasticizer/modifier provided the efficient extraction of vanadium(V) (initial concentration 50 mg L^−1^) from 0.1 M sulfate solutions (pH 2.5). The average mass and thickness of the PIMs (diameter 3.5 cm) were 0.057 g and 46 μm, respectively. It was suggested that V(V) was extracted as VO_2_SO_4_^−^ via an anion exchange mechanism. The maximum PIM capacity was estimated to be ~56 mg of V(V)/g for the PIM. Quantitative back-extraction was achieved with a 50 mL solution of 6 M H_2_SO_4_/1 *v*/*v*% of H_2_O_2_. It was assumed that the back-extraction process involved the oxidation of VO_2_^+^ to VO(O_2_)^+^ by H_2_O_2_. The newly developed PIM, with the optimized composition mentioned above, exhibited an excellent selectivity for V(V) in the presence of metallic species present in digests of spent alumina hydrodesulfurization catalysts. Co-extraction of Mo(VI) with V(V) was eliminated by its selective extraction at pH 1.1. Characterization of the optimized PIM was performed by contact angle measurements, atomic-force microscopy, energy dispersive X-ray spectroscopy, thermogravimetric analysis/derivatives thermogravimetric analysis and stress–strain measurements. Replacement of dibutyl phthalate with *2*-nitrophenyloctyl ether improved the stability of the studied PIMs.

## 1. Introduction

Vanadium, with some unique properties including hardness, fatigue resistance, tensile strength, good corrosion resistance at low temperatures and a high melting point [1], is a valuable metal used extensively in a variety of industrial applications such as manufacturing of electronic equipment and automobiles, nuclear reactor construction, glass coating processes, and catalyst production [2,3]. It is estimated that vanadium forms 0.019% of the Earth’s crust, being its eighteenth most abundant element [4]. Nevertheless, the great number of industrial needs and the increasing consumption of this element, on one hand, and the depletion of the corresponding mineral resources, on the other, require the development of efficient methods for the recovery of vanadium from second-hand sources and industrial waste [5]. Moreover, vanadium is considered as a serious contaminant [6], similarly to mercury, lead, and arsenic [7]. The International Agency for Research on Cancer has reported that vanadium is possibly carcinogenic to humans [8]. Among the vanadium species with different oxidation states, its pentavalent oxidation state is more noxious than the others [6]. Thus, vanadium pollution of environmental waters due to discharging improperly managed industrial wastes is of considerable concern, which, together with the associated economic benefits, justifies the recovery of vanadium from industrial wastes [9] and its clean-up from contaminated waters [10].

Although solid-phase extraction of vanadium using metal oxides [11] and modified chitosan [12] as adsorbents has shown some success in the vanadium clean-up of contaminated waters, solvent extraction has evolved as the most popular technique for vanadium separation [13,14]. This separation technique is relatively simple to execute, rapid, applicable to large-scale separation processes and highly selective, while at the same time it does not require sophisticated equipment [15]. However, industrial solvent extraction uses large volumes of volatile, flammable and toxic diluents, as well as a significant amount of energy, which is of considerable safety and environmental concern [16].

Separation that is based on the use of liquid membranes offers an attractive alternative to solvent extraction applications in both chemical analysis and industrial production [17]. Bulk liquid membranes (BLMs), emulsion liquid membranes (ELMs) and supported liquid membranes (SLMs) have been studied extensively for their potential in industrial separation and water treatment [18]. The limited interfacial surface area and the resulting relatively slow mass transfer rates are the main disadvantages of separations based on BLMs. The limitations in the use of ELMs are caused mainly by difficulties associated with the formation and breakdown of the double emulsion used. Relatively poor long-term stability caused by leaking of the extractant solution into the adjacent aqueous phases is the main drawback of SLMs [19].

Polymer inclusion membranes (PIMs) are a relatively new type of liquid membranes which are composed of a base polymer and an extractant, often referred to as a carrier [20]. In some cases, a plasticizer/modifier may also be present in the PIM composition. To enhance the performance of PIMs, some other miscellaneous strategies have been also employed. Among these strategies are the improvement of mechanical properties of PIMs by addition of montmorillonite (MMT) [21], and the enhancement of the extraction capacity of PIMs by using nanoparticles [22].

PIMs are visibly similar to SLMs, but they have a different structure, i.e., the extractant is located within a network of nanometer-sized channels in PIMs, while it is retained by relatively weak capillary forces within micrometer size pores of SLMs, thus leading to poorer long-term stability [19]. The performance of a PIM may be improved by selecting an optimized composition of its constituents. In most cases such optimization is performed by the univariate method. Besides, the potential of the response surface methodology (RSM) for the optimization of PIMs has been also demonstrated [23].

Although the introduction of PIMs as the sensing membranes in ion-selective electrode dates back more than 50 years, PIM applications in industrial and analytical separation have attracted significant interest only for the past decade [24,25].

Aliquat^®^ 336 is an anionic liquid containing a mixture of quaternary alkylammonium chlorides, with trioctylmethylammonium chloride (called also tricaprylmethylammonium chloride) being the main component. The potential of Aliquat^®^ 336 as an anionic extractant has been utilized in a variety of separation techniques, including solvent extraction methods [26], and those based on the use of BLMs [27], ELMs [28], SLMs [29], and PIMs [25].

The suitability of Aliquat^®^ 336 for the extraction-based separation of V(V) has been already demonstrated [30]. El-Nadi et al. have reported on the extraction of vanadium from acidic and alkaline media using Aliquat^®^ 336 dissolved in kerosene containing 10% n-octanol as phase modifier. The results indicated that the acidic route of leaching and extraction leads to avoiding the complication of the existence of molybdenum as an interfering metal [31]. The results of this study have indicated that the acidic route of leaching and extraction eliminates the co-extraction of molybdenum. In a previous study we achieved the selective separation of V(V) from Mo(VI) by using a PIM composed of poly(vinylidenefluoride-*co*-hexafluoropropylene) (PVDF-HFP) as the base polymer, trihexyltetradecylphosphonium chloride (Cyphos^®^ IL 101) as the extractant and *2*-nitrophenyloctyl ether (NPOE) as a plasticizer/modifier [32]. However, both the extractant and the plasticizer/modifier of this PIM are relatively expensive. Thus, the present reports on the development of a method for the separation of V(V) from its sulfate solutions using a PVDF-HFP-based PIM, containing the less expensive extractant and plasticizer/modifier Aliquat^®^ 336 and dibutyl phthalate, respectively. To the best of the author’s knowledge this is the first use of this extractant and plasticizer/modifier in a polymer inclusion membrane for the extraction of V(V).

## 2. Methods

### 2.1. Reagents

Aliquat^®^ 336 (≥98%, Aldrich, Burlington, MA, USA), PVDF-HFP (Aldrich, Burlington, MA, USA), HPLC grade tetrahydrofuran (THF) (99.9%, Samchun, Pyeongtaek, South Korea), 2-nitrophenyloctyl ether (NPOE) (>99%, Fluka, Switzerland), tributylphosphate (TBP) (>98%, Merck, Darmstadt, Germany), tris(2-ethylhexyl)phosphate (TEHP) (≥98%, Merck, Darmstadt, Germany), dibutyl phthalate (DBP) (99%, Merck, Darmstadt, Germany) and reduced graphene oxide nanoplatelets (rGONPs) (Green Nanoscale Technology, Mashhad, Iran) were used in the PIM preparation. Sodium orthovanadate (99.98%, Sigma-Aldrich, Burlington, MA, USA), hydrogen peroxide (36%, Dr. Mojallali, Iran), barium chloride dihydrate (≥99%, Merck, Darmstadt, Germany), sodium carbonate (≥99.9%, Merck, Darmstadt, Germany), sodium sulfate anhydrous (≥99%, Dr. Mojallali, Tehran, Iran), sulfuric acid (98%, Merck, Darmstadt, Germany), hydrochloric acid (37%, Dr. Mojallali, Iran), nitric acid (65%, Merck, Darmstadt, Germany) and sodium hydroxide (≥95%, Dr. Mojallali, Tehran, Iran) were utilized in the preparation of the solutions employed in the extraction and back-extraction experiments. Aluminum chloride (≥98%, Merck, Darmstadt, Germany), manganese(II) nitrate tetrahydrate (≥98.5%, Merck, Darmstadt, Germany), nickel(II) nitrate hexahydrate (≥96%, Fluka, Buchs, Switzerland), copper(II) nitrate trihydrate (≥99.5%, Merck, Darmstadt, Germany), iron(III) nitrate (≥99%, Merck, Darmstadt, Germany), cobalt(II) nitrate hexahydrate (≥99%, Merck, Darmstadt, Germany) and sodium molybdate(VI) dihydrate (≥99%, Acros, Branchburg, NJ, USA) were used in investigating PIM selectivity. Xylenol orange (Merck, Darmstadt, Germany), used as the colorimetric reagent for the analysis of V(V), was dissolved in an acetate buffer solution. The buffer solution was prepared by using glacial acetic acid (≥99.8%, Dr. Mojallali, Tehran, Iran). Deionized water (resistivity ≥ 18.2 MΩ cm, Zolalan, m-uv-3^+^, Iran) was used for the preparation of all aqueous solutions.

### 2.2. Instrumentation

A circulating water bath fitted with a digital thermoregulator (Org Mp-5, Julabo, Seelbach, Germany) was used for keeping the temperature constant during the dissolution of the PIM components in THF. Membrane casting solutions were stirred using a magnetic stirrer (IKA, Staufen, Germany). A platform orbital shaker (PIT 10 LO, PIT, Iran) was employed for shaking the source and back-extraction solutions during the extraction and back-extraction experiments. The pH measurements were done with a glass electrode (Metrohm, Switzerland) connected to a pH meter (780, Metrohm, Herisau, Switzerland). A UV–visible spectrophotometer (DR5000, Hach, Loveland, CO, USA) was used for the detection of the V(V)-xylenol orange complex. Flame atomic absorption spectrometry (FAAS) with a nitrous oxide/acetylene flame (novAA 350, Analytic Jena, Göttingen, Germany) was employed for the analysis of vanadium in the cases that the chemical composition of the samples prohibited the determination of V(V) by the spectrophotometric method mentioned above. Membrane thickness measurements were made using a caliper (SL-M, Insize, Suzhou, China). The homogeneity of the PIMs was characterized by energy dispersive X-ray spectroscopy (TESCAN mira3, Brno, Czech Republic). An atomic force microscope (AFM) (Nano Vac, Ara Research Company, Tehran, Iran), operated in contact mode, was used for studying membrane surface morphology. Thermogravimetric analysis (TGA) and derivatives thermogravimetric analysis (DTGA) were conducted by using a STA 409 PC/PG analyzer (Netzsch, Selb, Germany). A contact angle goniometer (CAG-10, Jikan, Tehran, Iran) was employed for the contact angle measurements. The stress-strain behavior of the PIMs was investigated by using a force digital gauge (STM-5 Cap. 5 kN, Santam, Tehran, Iran) connected to a personal computer. The samples studied were 1 cm in width and 5 cm in length. All the measurements were performed at a strength rate of 50 mm min^−1^.

### 2.3. Membrane Preparation

PIMs were prepared by dissolving 1.0 g PVDF-HFP, Aliquat^®^ 336 and plasticizer/modifier in 10 mL of THF (10 mL of THF per 1 g of the polymer PVDF-HFP). The mixture was magnetically stirred for 2 h at room temperature (22 ± 1 °C), followed by a further 2 h of stirring at 40 °C. The solution was poured into a homemade Teflon casting knife [33], placed on a glass plate. The casting knife was then displaced along the glass plate to form a thin layer of the membrane casting solution. The glass plate was covered with an aluminum tray to allow slow the evaporation of THF for 24 h. Circular membrane segments were cut from the casted PIM (using a 3.5 cm diameter steel punch) and used in the extraction and back-extraction experiments. The membrane, with an optimal composition (i.e., 50 wt% PVDF-HFP, 40 wt% Aliquat^®^ 336 and 10 wt% DBP), had an average mass of 0.057 ± 0.005 g and thickness of 46 ± 6 μm. The optimizations in this study have been based on the univariate method.

### 2.4. Extraction and Back-Extraction Experiments

**Figure 1:**
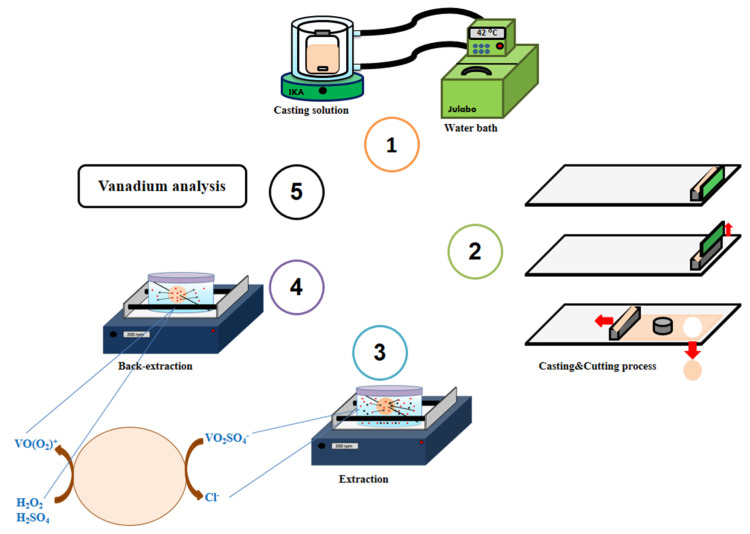
Schematic representation of the experimental procedure.

In the extraction experiments, circular PIM segments (3.5 cm in diameter) were immersed in 50 mL of solutions containing 9.8 × 10^−4^ mol L^−1^ (50 mg L^−^^1^) V(V) and 0.2 mol L^−1^ sulfate. The pH of these solutions was adjusted to 2.5 by adding sulfuric acid or sodium hydroxide solutions. The solutions containing the PIM were agitated on a platform orbital shaker (200 rpm). Sampling was performed by withdrawing 0.2 mL of solution at predetermined times during the experiments. The samples were diluted and analyzed either spectrophotometrically at 522 nm for the determination of V(V), with the complexing reagent xylenol orange, or by FAAS. A schematic representation of the experimental procedure is represented in Figure 1.

### 2.5. Eliminating the Co-Extraction Mo(VI) with V(V)

A two-step procedure developed in an earlier study [32] was applied for the removal of Mo(VI), which otherwise would be co-extracted with V(V). The first step involved the adjustment of the aqueous 0.2 mol L^−1^ sulfate solution, containing V(V), Mo(VI) Al(III), Co(II), Cu(II), Fe(III), Mn(II) and Ni(II) (50 mg L^−1^ each), to pH 1.1 and immersing an optimized PIM in this solution for 24 h under shaking. The PIM was then removed from the solution and immersed in 50 mL of 6 mol L^−1^ H_2_SO_4_ containing 1 *v*/*v*% H_2_O_2_ for the recovery of the extracted metallic species. In the second step of the approach, the solution pH was increased to 2.5 and a fresh 3.5 cm circular PIM was immersed in it for 24 h under shaking. This was followed by withdrawing the PIM from the solution, rinsing it with deionized water and immersing it in 50 mL of 6 mol L^−1^ H_2_SO_4_ solution containing 1 *v*/*v*% of H_2_O_2_.

## 3. Results and Discussion

### 3.1. Preliminary Extraction Experiments

**Figure 2:**
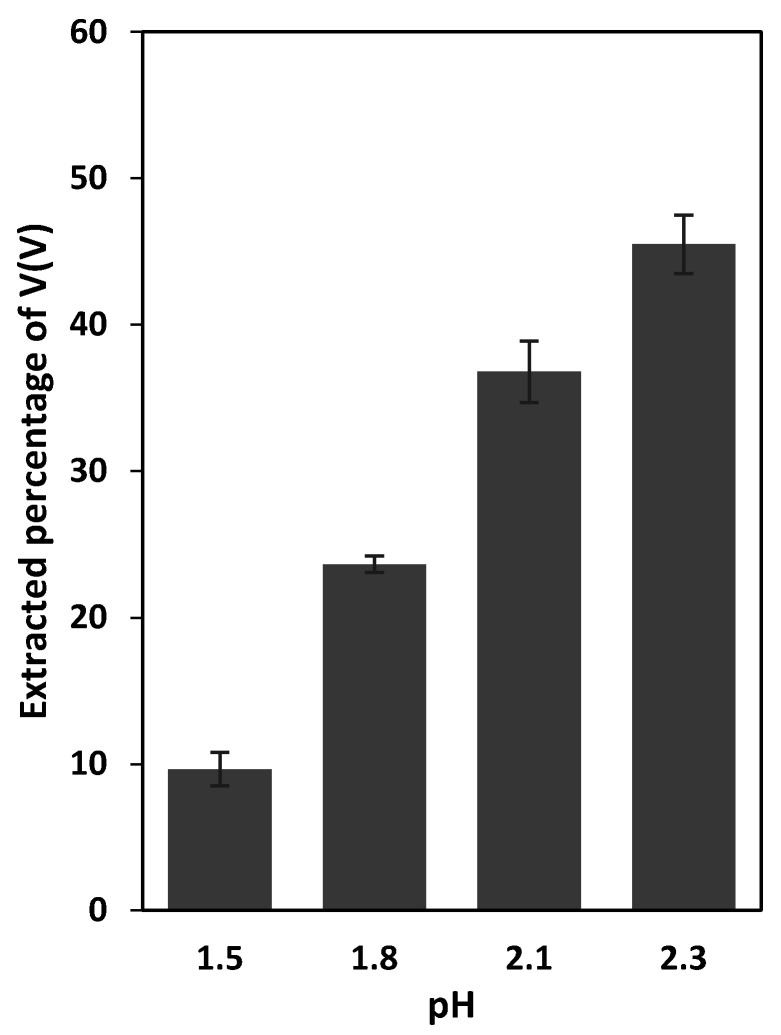
Effect of the aqueous source solution pH on the extraction of V(V) (initial concentration 50 mg L^−1^) from 0.2 mol L^−1^ sulfate solutions into PVDF-HFP-based PIMs containing 30 wt% Aliquat^®^ 336. PIMs/aqueous source solution contact time and temperature were 8 h and 22 ± 1 °C, respectively. Error bars = ±standard deviation (SD).

The variation of the extraction percentage of V(V) as a function of the aqueous source solution pH was investigated by performing a series of extraction experiments, using the PIMs composed of 70 wt% of PVDF-HFP and 30 wt% Aliquat^®^ 336. In these experiments, a circular PIM with 3.5 cm diameter was immersed in 50 mL of 0.2 mol L^−^^1^ sulfate solution containing 50 mg L^−1^ V(V) ions. The pH of the aqueous solutions was adjusted to 1.5, 1.8, 2.1, 2.3 or 2.7, and the extraction percentage of V(V) after 8 h was measured (Figure 2).

As expected, the extraction of V(V) was found to be pH-dependent. This may be interpreted by considering the pH-dependency of the distribution of the V(V) species (i.e., VO_2_^+^, VO_2_SO_4_^−^, H_2_VO_4_^−^, HVO_4_^2−^, H_2_V_10_O_28_^4−^, HV_10_O_28_^5−^, V_4_O_12_^4−^, HV_2_O_7_^3−^ and V_2_O_7_^4−^) [3].

The PIM in contact with the solution that was adjusted to pH 1.5 was colorless and transparent. Under such conditions, the extraction percentage of V(V) was lower than 10%. When the solution pH was increased to 1.8 and 2.1, the PIMs became brown but remained transparent. The extraction percentages of V(V) from such solutions were around 24 and 37%, respectively. The transparency of the PIMs at the pH mentioned above at extraction equilibrium indicated that the membranes were homogeneous and thus compatible with the extracted complex.

By increasing the pH to 2.3, the increase in the extraction percentage of V(V) caused the opaqueness of the PIMs. This observation was attributed to the low solubility of the extracted V(V)-Aliquat^®^ 336 adduct in the PIM liquid phase. A further increase in pH to 2.7 resulted in the formation of yellow-colored sediment on the surface of the PIMs, which was most likely polyoxovanadate [3].

### 3.2. Selection of a Plasticizer/Modifier

**Figure 3:**
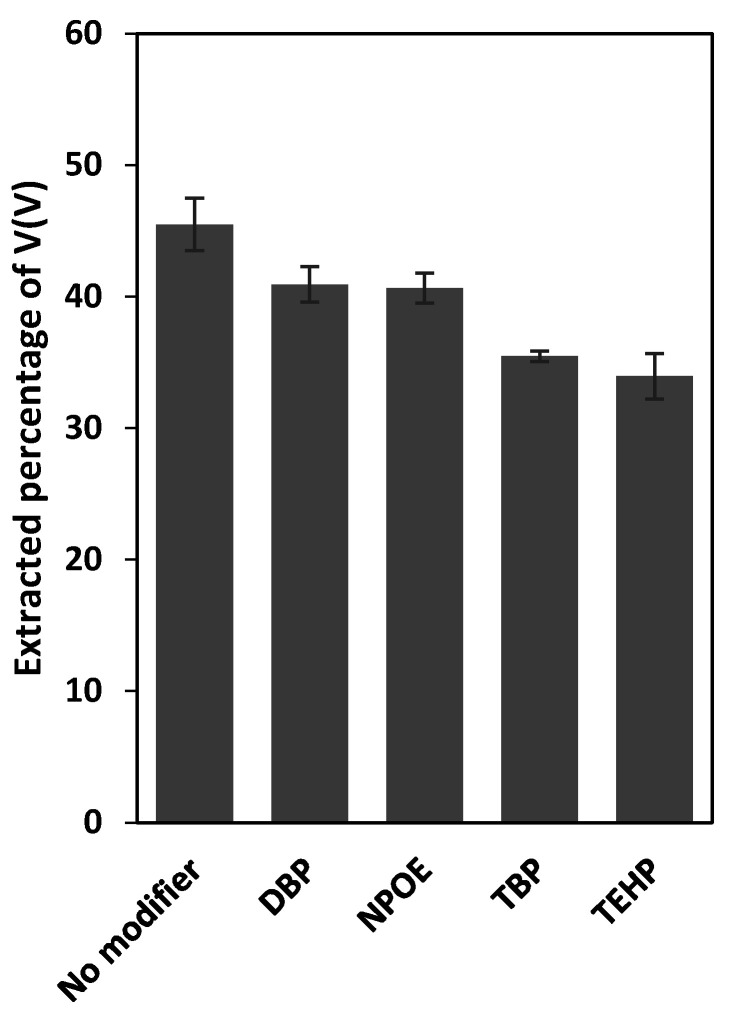
Effect of the plasticizer/modifier on the extraction of V(V) from aqueous 0.2 mol L^−1^ sulfate solutions, adjusted to pH 2.3 and containing initially 50 mg L^−1^ V(V) into PVDF-HFP-based PIMs incorporating either 30 wt% Aliquat^®^ 336 and no plasticizer/modifier or 25 wt% Aliquat^®^ 336 and 5 wt% plasticizer/modifier (DBP, NPOE, TBP or TEHP). PIMs/aqueous source solution contact time and temperature were 8 h and 22 ± 1 °C, respectively. Error bars = ±SD.

The improvement of the compatibility of the membrane components and/or improvement in the solubility of the extracted adduct into the membrane liquid phase are the main roles attributed to the plasticizer/modifier in a PIM [34]. Therefore, the effect of several plasticizers/modifiers including NPOE, DBP, TEHP and TBP on reducing/eliminating the incompatibility issues mentioned above was studied and the results are presented in Figure 3.

The results revealed that all four plasticizer/modifiers eliminated the limited solubility of the V(V)- Aliquat^®^ 336 adduct in the corresponding PIMs. The higher extraction percentage of V(V) by the plasticizer/modifier-free PIM, compared to that of four plasticizer/modifier-containing PIMs (Figure 2), was attributed to their lower concentration of Aliquat^®^ 336, i.e., 25 wt%, as opposed to 30 wt% in the plasticizer/modifier-free PIMs. The results showed also that the extraction percentage of V(V) in the PIMs containing a plasticizer/modifier decreased in the order DBP ≈ NPOE > TBP > TEHP. Although the interpretation of the observed order is not straightforward, one should consider the complex effects of the viscosity and polarity of the plasticizers on the extraction results. Taking into account the similar extraction results for the PIMs containing NPOE or DBP, and the higher cost of NPOE, DBP was selected as the PIM plasticizer/modifier for the subsequent experiments.

### 3.3. Optimization of the PIMs Composition

To determine the optimal PIM composition, a series of PIMs with different concentrations of their components were prepared. These PIMs covered the ranges of 50–90 wt% PVDF-HFP, 10–50 wt% Aliquat^®^ 336 and 0–20 wt% DBP. It was found that PIMs containing more than 15 wt% DBP were sticky and mechanically weak. In addition, PIMs with extractant and polymer concentrations of greater than 40 and 55wt%, respectively, were also discarded, because while being initially transparent, they became cloudy and opaque when used in a single extraction experiment. This indicates the incompatibility of the extracted species with the other PIM constituents. The PIMs which were transparent, homogeneous, flexible and mechanically stable (Table 1) were deemed as successful and were examined in the extraction experiments.

All four successful PIMs did not change their physical properties as a result of the extraction experiments. Therefore, the PIM with the composition of 50 wt% PVDF-HFP, 40 wt% Aliquat^®^ 336 and 10 wt% DBP was selected as the best performing PIM for the subsequent experiments.

### 3.4. Effect of the Aqueous Solution pH on the Extraction Rate of V(V)

**Figure 4:**
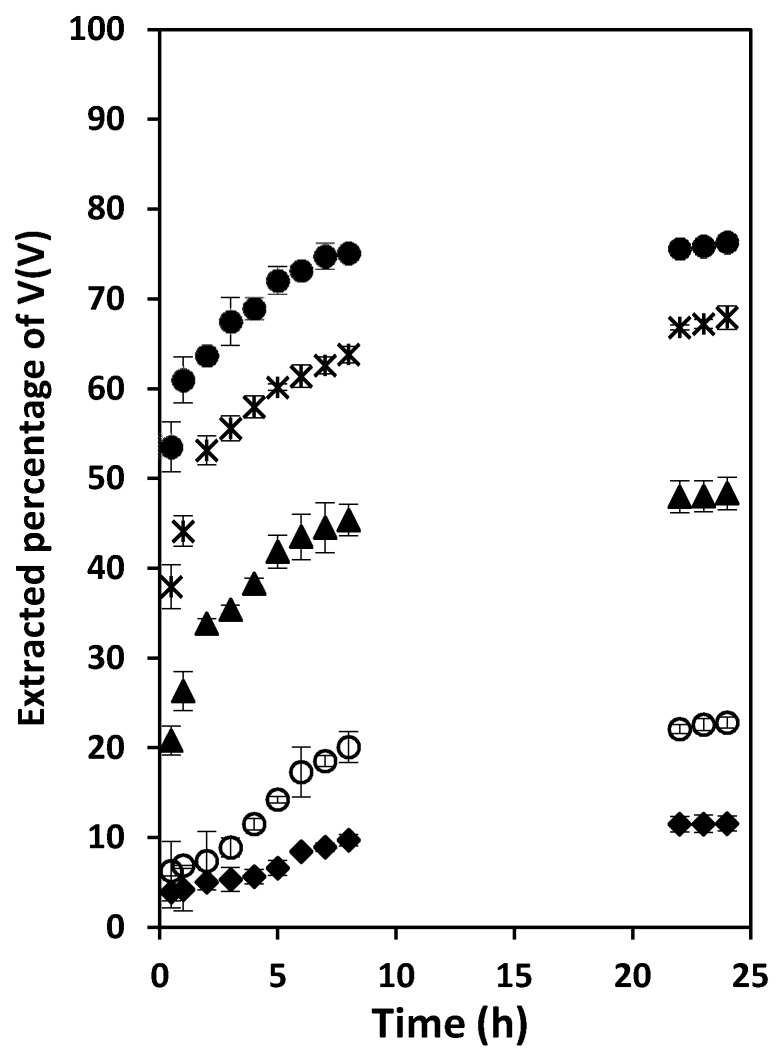
Variation of the extraction percentage of V(V) during its extraction from aqueous solutions adjusted to pH 1 ◆, 1.5 ○, 2.0 ▲, 2.3 ✴, or 2.5 ●. Experimental conditions: aqueous solution composition − 50 mg L^−1^ V(V) and 0.2 mol L^−1^ sulfate; PIM composition 50 wt% PVDF-HFP, 40 wt% Aliquat^®^ 336 and 10 wt% DBP; PIMs/aqueous source solution contact time and solution temperature 24 h and 22 ± 1 °C, respectively. Error bars = ± SD.

Based on the results obtained in the preliminary experiments, the pH of the source solution varied between 1 and 2.5. Figure 4 shows its effect on the extraction of V(V) by the PIM composed of 50 wt% PVDF-HFP, 40 wt% Aliquat^®^ 336 and 10 wt% DBP. The results confirmed that the extracted amount of V(V) across 24 h increased from 11.6 to 76.3% as the solution pH was raised from 1 to 2.5, respectively. For pH values above 2.5 the V(V)- Aliquat^®^ 336 started precipitating. Therefore, pH 2.5 was selected as the optimal pH for the source solution. It should be noted that VO_2_SO_4_^−^ is the dominant vanadium species in the source solution under the experimental conditions (i.e., pH 1–3 and V(V) concentration in the order of 10^−4^ mol L^−^^1^) [3]. An interpretation of the pH-dependency of V(V) extraction is given in Section 3.1.

### 3.5. Effect of the Sulfate ion Concentration and Characterization of the Extracted Species

Since VO_2_SO_4_^−^ is the dominant V(V) species present in the aqueous source solution under the selected experimental conditions, it was expected that the extraction of the V(V) follows the anion exchange mechanism described by Equation (1) [35,36].

VO_2_SO_4_^−^_(aq)_ + R_3_R’N^+^Cl^−^_(PIM)_ ⇆ [R_3_R’N^+^•VO_2_SO_4_^−^]_(PIM)_ + Cl^−^_(aq)_ (1)

where R_3_R’N^+^Cl^−^ denotes Aliquat^®^ 336.

As the sulfate anion is involved in the extraction of V(V), its effect was evaluated by varying its source solution concentration (i.e., 0.03, 0.05, 0.1, 0.2 and 0.3 mol L^−1^). A yellow precipitate was formed on the surface of the PIMs when the sulfate concentration was lower than 0.1 mol L^−1^. The yellow precipitate was most likely the result of polyoxovanadate formation at these low concentrations of the sulfate ion. The extraction of V(V) was found to be independent of the sulfate concentration when it was higher than 0.1 mol L^−^^1^, which was in agreement with the results of an earlier study [32]. Therefore, the subsequent extraction experiments were carried out by adjusting the sulfate ion concentration and the pH of the aqueous solutions to 0.2 mol L^−1^ and 2.5, respectively.

**Figure 5:**
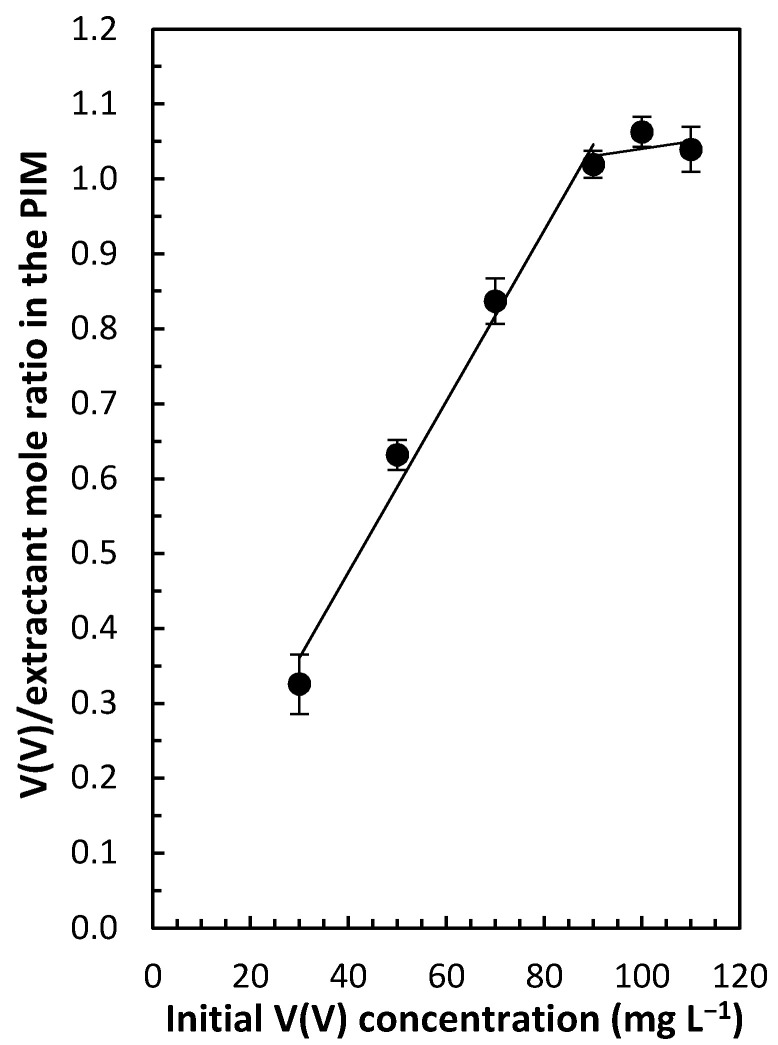
The variation of V(V)/Aliquat^®^ 336 mole ratio in the PIM studied (50 wt% PVDF-HFP, 40 wt% Aliquat^®^ 336 and 10 wt% DBP) as a function of the initial V(V) concentration in the aqueous source solution, containing 0.2 mol L^−1^ sodium sulfate and adjusted to pH 2.5. PIMs/aqueous source solution contact time and temperature were 24 h and 22 ± 1 °C, respectively. Error bars = ± SD.

The stoichiometry of the extracted V(V) adduct (Equation (1)) was confirmed by performing a series of extraction experiments on V(V) from source solutions adjusted to pH 2.5 and containing V(V) in the concentration range of 30–110 mg L^−^^1^, as well as 0.2 mol L^−1^ sulfate. The results presented in Figure 5 indicate that the PIM was saturated with V(V) when the mole ratio V(V)/Aliquat^®^ 336 approached one, which was in agreement with Equation (1). The calculations based on the results presented in Figure 5, along with the average PIM mass (0.057 g), allowed for the determination of the PIM extraction capacity as being 56 mg V(V) per 1 g of PIM.

### 3.6. Back-Extraction Studies

**Figure 6:**
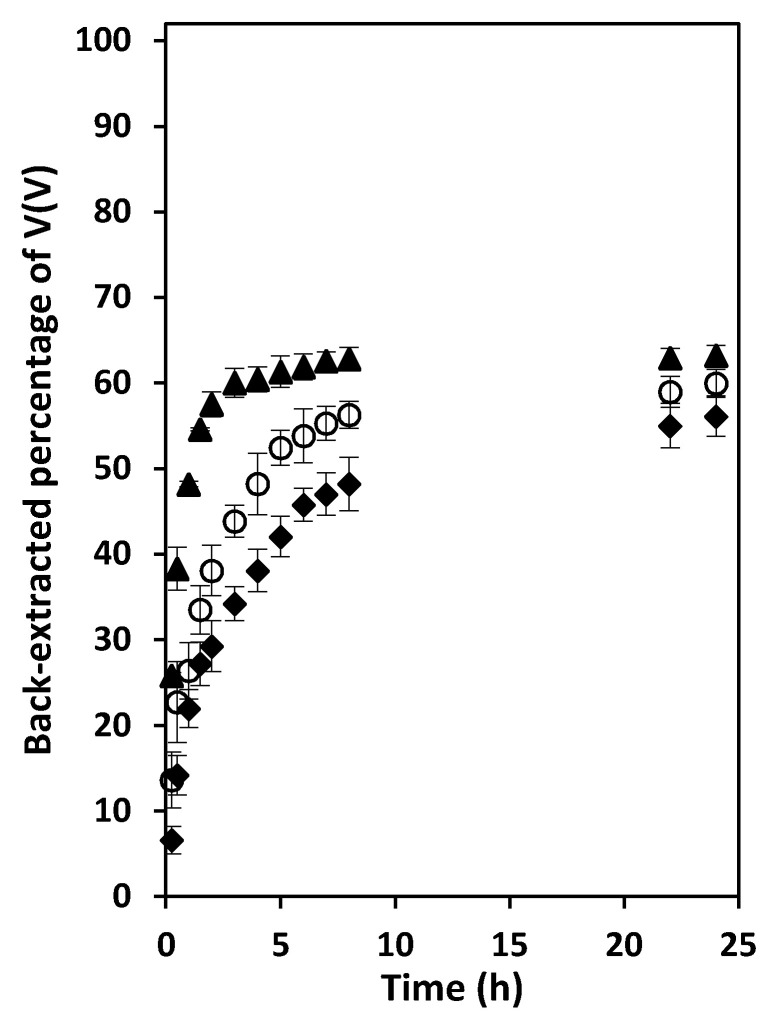
Back-extraction percentage of V(V) from loaded PIMs (50 wt% PVDF-HFP, 40 wt% Aliquat^®^ 336 and 10 wt% DBP; 33.5 mg V(V)/g PIM) in solutions containing 1.0 (◆), 3.0 (○) or 6.0 (▲) mol L^−^^1^ sulfuric acid under shaking as a function of time. PIMs/aqueous back-extraction solution contact time and temperature were 24 h and 22 ± 1 °C, respectively. Error bars = ± SD.

The average percentage of V(V) extracted after 24 h into the best performing PIM (#4, Table 1), from aqueous source solutions containing 50 mg L^−1^ V(V) and adjusted to pH 2.5, was determined to be 76.3 ± 0.5%, which corresponded to 60% saturation with respect to the moles of extractant in the PIM. A back-extraction study, in which V(V)-loaded PIMs were immersing for 24 h in 1 mol L^−1^ solutions of hydrochloric acid, nitric acid or sulfuric acid under shaking, produced back-extraction percentages of 44.6 ± 0.9, 44.4 ± 0.8 and 56.1 ± 1.1%, respectively. Although the back-extraction of V(V) was relatively more successful by using the sulfuric acid solution, none of the tested stripping reagents were able to provide a quantitative V(V) back-extraction. Therefore, back-extraction experiments with higher concentrations of sulfuric acid were conducted, which demonstrated that an increase in the sulfuric acid concentration increased both the back-extraction percentage and its rate (Figure 6). However, quantitative back-extraction of V(V) could not be achieved even in the case when a 6 mol L^−1^ sulfuric acid solution was used.

The possibility of improving the efficiency of back-extraction by converting the back-extracted VO_2_^+^ species to the oxoperoxo species VO(O_2_)^+^ in the presence of H_2_O_2_ (Equation (2), [37]) was explored by adding H_2_O_2_ to the sulfuric acid back-extraction solution.

VO_2_^+^ + H_2_O_2_ → VO(O_2_)^+^ + H_2_O (K = 3.5 × 10^4^ at 25 °C) (2)

The overall back-extraction process in this case can be described by Equation (3).

VO_2_SO_4_^−^_(PIM)_ + H^+^_(aq)_ + H_2_O_2 (aq)_ → VO(O_2_)^+^_aq_ + HSO_4_^−^_(PIM)_ + H_2_O_(aq)_ (3)

where the subscripts “aq” and “PIM” denote “aqueous” or “PIM”, respectively.

**Figure 7:**
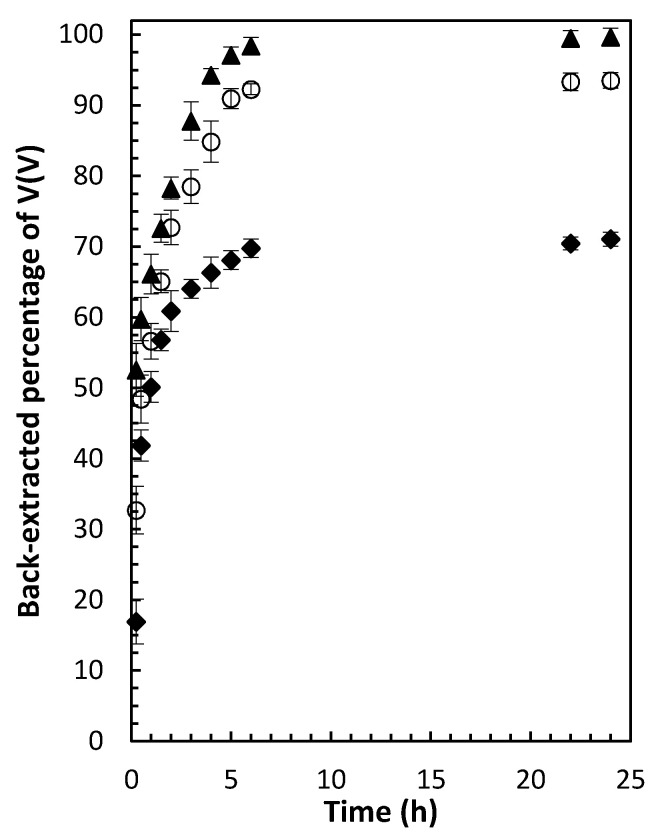
Back-extracted percentage of V(V) from PIMs (50 wt% PVDF-HFP, 40 wt% Aliquat^®^ 336 and 10 wt% DBP, 33.5 mg V(V)/g PIM) using sulfuric acid solutions (1.0 ◆, 3.0 ○ and 6.0 ▲ mol L^−^^1^) containing 1 *v*/*v*% H_2_O_2_ as a function of time. PIMs/aqueous back-extraction solution contact time and temperature were 24 h and 22 ± 1 °C, respectively. Error bars = ± SD.

The results when the back-extraction solution contained 1 mol L^−1^ sulfuric acid and 1 *v*/*v*% H_2_O_2_ showed that the presence of hydrogen peroxide increased the V(V) back-extraction percentage from 56.1 ± 0.4 to 71.1 ± 0.4%. Increasing the concentration of sulfuric acid to 3 and 6 mol L^−1^ while maintaining the H_2_O_2_ concentration at 1 *v*/*v*% further enhanced back-extraction efficiency, reaching complete back-extraction at 6 mol L^−1^ (Figure 7).

**Figure 8:**
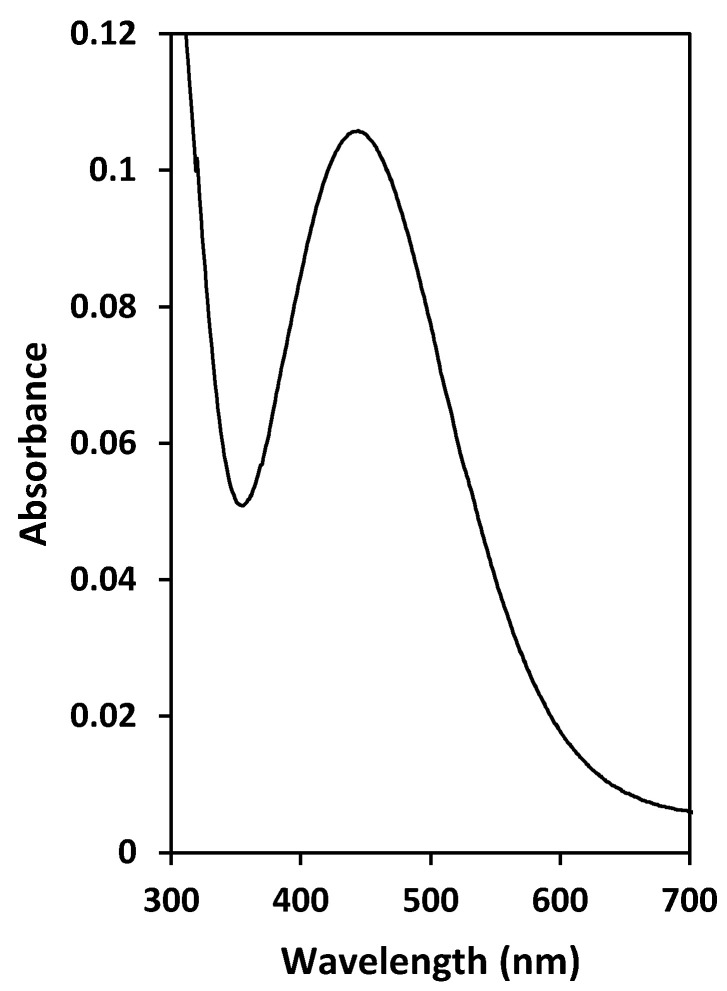
UV-Vis spectrum of the back-extraction solution (6 mol L^−1^ H_2_SO_4_ + 1 *v*/*v*% H_2_O_2_), confirming the presence of the VO(O_2_)^+^ species [38].

It should be noted that the back-extraction solution was reddish-brown (Figure 8), thus confirming the presence of the VO(O_2_)^+^ species [38]. To confirm the presence of the sulfate ion in the PIM as a result of the back-extraction process described by Equation (3), a back-extracted PIM was washed with deionized water and then immersed in a dilute solution of BaCl_2_. The formation of the white BaSO_4_ precipitate on the PIM surface indicated the presence of sulfate species in the PIM, which were exchanged for the chloride ions of the BaCl_2_ reagent.

### 3.7. PIM Selectivity

The optimized PIM (50 wt% PVDF-HFP, 40 wt% Aliquat^®^ 336 and 10 wt% DBP) was assessed for its selectivity in the extraction of V(V) from solutions containing Mo(VI), Al(III), Co(II), Cu(II), Fe(III), Mn(II) and Ni(II) species, which are usually present in the digests of spent hydrodesulfurization catalysts. Experiments involving the extraction of V(V) from solutions containing only one or all of these metallic species showed that, except for Mo(VI), the newly developed PIM was highly selective for V(V) (Table 2). These results are in agreement with those of a previous study, where a PIM composed of PVDF-HFP, Cyphos^®^ IL 101 (trihexyltetradecylphosphonium chloride) and NPOE, as the base polymer, extractant, and plasticizer/modifier, respectively, was applied for the extraction of V(V) from solutions of similar compositions [32].

The co-extraction of Mo(VI) from Solution 2 (Table 2) decreased the extraction percentage of V(V) by approximately 24%. Therefore, under the selected experimental conditions, the PIM could only be directly applied for the separation of V(V) from samples which did not contain Mo(VI). Therefore, it was necessary to remove Mo(VI) from the solution before using the PIM for the extraction of V(V). A two-step procedure developed in an earlier study [32] and outlined earlier was applied. The first step resulted in the quantitative removal of Mo(VI), while V(V) was selectively extracted in the second step. The results, presented in Table 3, indicate that the two-step procedure allowed for the selective separation of V(V) from the initial solution containing Mo(VI), Al(III), Co(II), Cu(II), Fe(III), Mn(II) and Ni(II).

### 3.8. PIM Reusability and Stability

**Figure 9:**
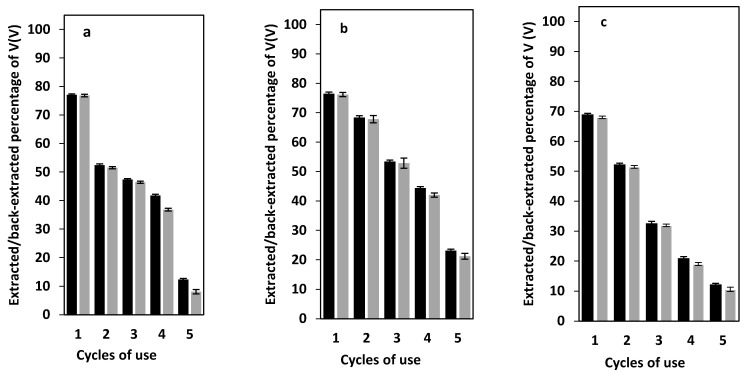
Extraction (black bars)/back-extraction (grey bars) percentage of V(V) in the case of PIMs containing (**a**) 50/40/10 wt% of PVDF-HFP/Aliquat^®^ 336/DBP, (**b**) 50/40/10 wt% of PVDF-HFP/Aliquat^®^ 336/NPOE, or (**c**) 49/40/10/1 wt% of PVDF-HFP/Aliquat^®^ 336/DBP/rGONPs. Experimental conditions: aqueous source solution 50 mL of 50 mg L^−1^ V(V) and 0.2 mol L^−1^ sulfate ion (pH 2.5), back-extraction solution 50 mL of 6 mol L^−1^ H_2_SO_4_ and 1 V/V% H_2_O_2_. PIMs/aqueous solutions shaking time in both the extraction and back-extraction processes 24 h.

**Figure 10:**
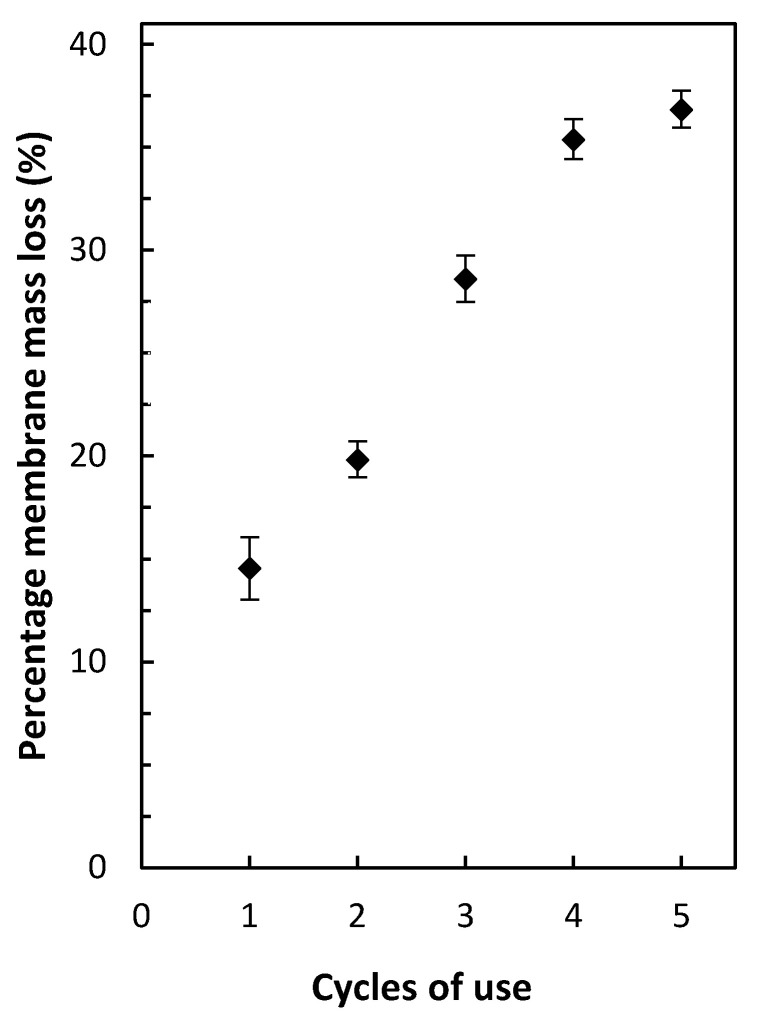
Percentage mass loss of PIMs containing PVDF-HFP/Aliquat^®^ 336/DBP 50/40/10 wt% after each extraction/back-extraction cycle. Error bars = ±SD.

The reusability of the newly developed PIM was evaluated by conducting five consecutive extraction/back-extraction cycles (24 h each) (Figure 9a). It was observed that the extraction efficiency of the PIM used in the second extraction/back-extraction cycle dropped ~24%, with respect to its extraction efficiency in the first cycle. The extraction efficiency further decreased with each subsequent extraction/back-extraction cycle. The most likely reason for this effect was the leaking of the membrane liquid phase into the aqueous solutions that were in contact with the PIM, as observed in other studies [39], which was confirmed by the membrane mass loss after each extraction/back-extraction cycle (Figure 10).

By assuming that the plasticizer/modifier influences the PIMs characteristics [40], DBP in the investigated PIM was replaced by NPOE to prepare a PIM composed of PVDF-HFP/Aliquat^®^ 336/NPOE (50/40/10 wt%). Since it has been reported that the presence of reduced graphene oxide nanoparticles (rGONPs) may improve the stability of PIMs [41], PIMs containing PVDF-HFP/Aliquat^®^ 336/DBP/rGONPs (49/40/10/1 wt%) were also prepared. The reusability of these PIMs was compared with that of the original DBP-based PIM. The results presented in Figure 9a–c revealed that the replacement of DBP by NPOE improved, to some extent, the stability of the PIM, unlike the addition of rGONPs. Comparison of these results with those reported on the extraction of V(V) by a PIM containing Cyphos^®^ IL 101 [32] showed better stability for the Cyphos^®^ IL 101-based PIM, due to the lower water solubility of Cyphos^®^ IL 101 when compared to Aliquat^®^ 336.

### 3.9. PIM’s Characterization

**Figure 11:**
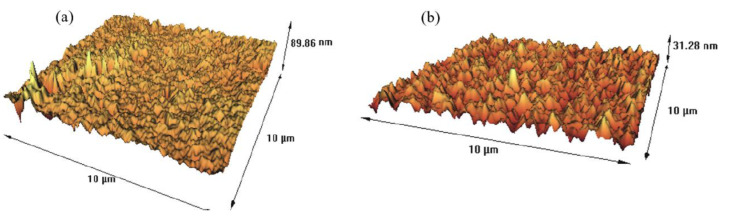
AFM three dimensional topographic images of the surface of (**a**) the blank PVD-HFP film and (**b**) the optimized PIM consisting of 50 wt% PVDF-HFP, 40 wt% Aliquat^®^ 336 and 10 wt% DBP.

To evaluate the hydrophobicity of the optimized PIM, its contact angle was measured and compared with that of a blank PVDF-HFP film. The contact angle of the blank PVDF-HFP film (94°) was considerably higher than that of the PVDF-HFP/Aliquat^®^ 336/DBP 50/40/10 wt% PIM (28°). The higher hydrophilicity of the optimized PIM could be attributed to the polar groups in both Aliquat^®^ 336 and DBP. The contact angle values agree with the results obtained in the AFM study of the optimized PIM and the blank PVDF-HFP film, where it was found that the roughness of the former (3.82 nm) was lower than that of the latter (19.8 nm) (Figure 11). It has been reported that rougher surfaces are characterized by higher contact angles [42]. The decreased roughness of the optimized PIM when compared to that of the blank PVDF-HFP film can be attributed to the presence of the membrane liquid phase [43].

**Figure 12:**
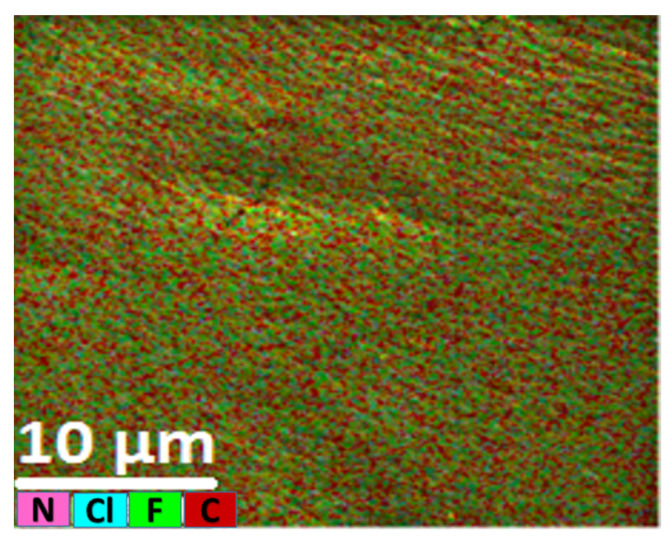
EDS-layered image and compositional map of the optimized PIM (50 wt% PVDF-HFP, 40 wt% Aliquat^®^ 336 and 10 wt% DBP).

Energy dispersive X-ray spectroscopy (EDS) can provide information about the uniformity of distribution of the extractant within the PIM. Figure 12, presenting an EDS image of the optimum PIM, indicates the uniform distribution of nitrogen and chlorine as the two main constituent elements of Aliquat^®^ 336, which are not encountered in the other membrane components.

**Figure 13:**
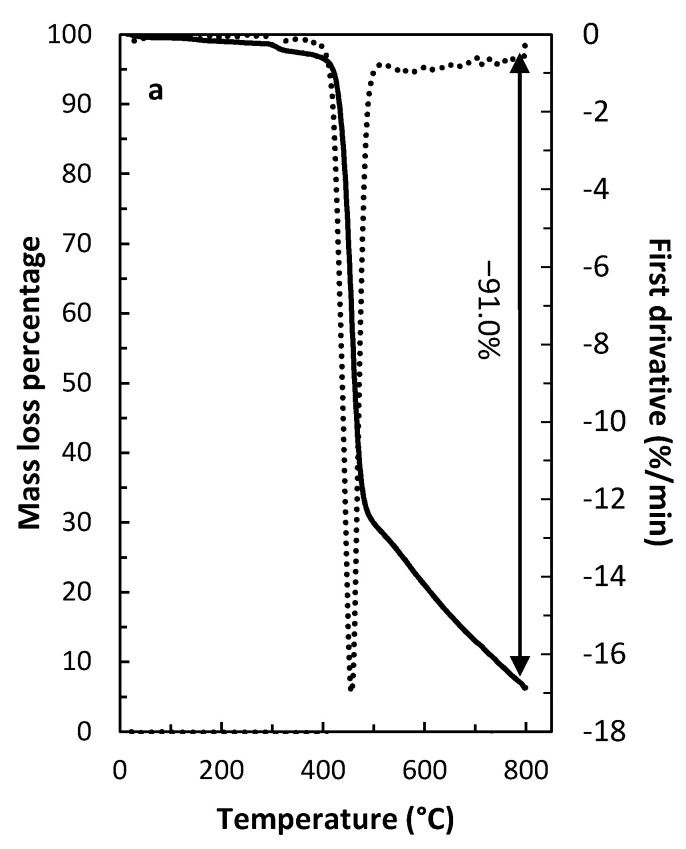

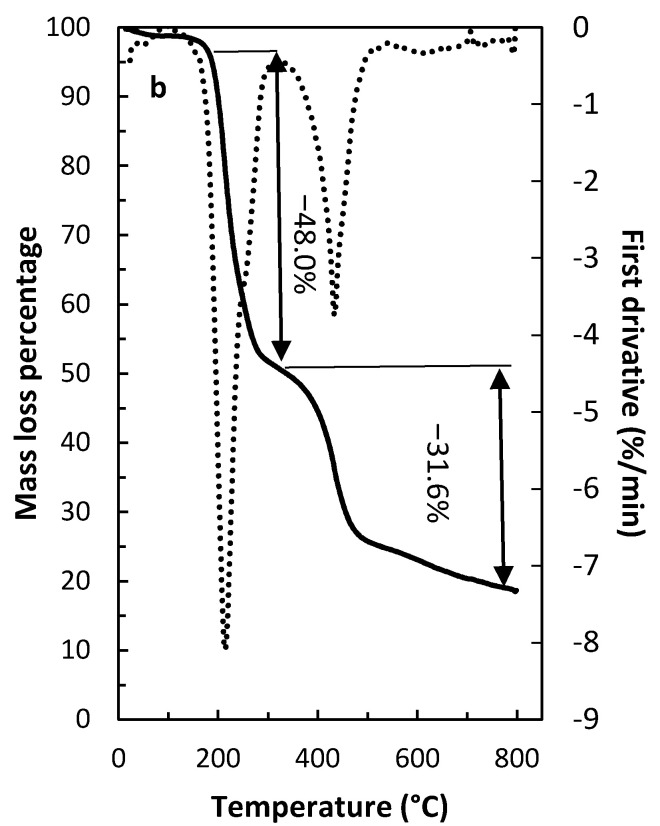
TGA and DTGA thermograms of (**a**) the PVDF-HFP film and (**b**) the optimized PIM (50 wt% PVDF-HFP, 40 wt% Aliquat^®^ 336 and 10 wt% DBP) obtained under N_2_ atmosphere.

Thermogravimetric analysis (TGA) and derivative thermogravimetric analysis (DTGA) results for the PVDF-HFP film and the optimized PIMs, presented in Figure 13, revealed that the blank PVDF-HFP underwent thermal decomposition under 450 °C in one single step, with a 91.0% mass loss (Figure 13a). However, the thermal decomposition of the optimized PIM proceeded in two main steps (Figure 13b). The first step, starting at approximately 170 °C, resulted in a 48.0% mass loss and was attributed to the loss of both extractant and plasticizer/modifier. The second step, starting at 330 °C, led to a 31.6% mass loss and corresponded to the decomposition of the base polymer. The shift of the PVDF-HFP decomposition to lower temperatures for the optimized PIM when compared to the blank PVDF-HFP film was most likely caused by interactions between the PIM components [44].

**Figure 14:**
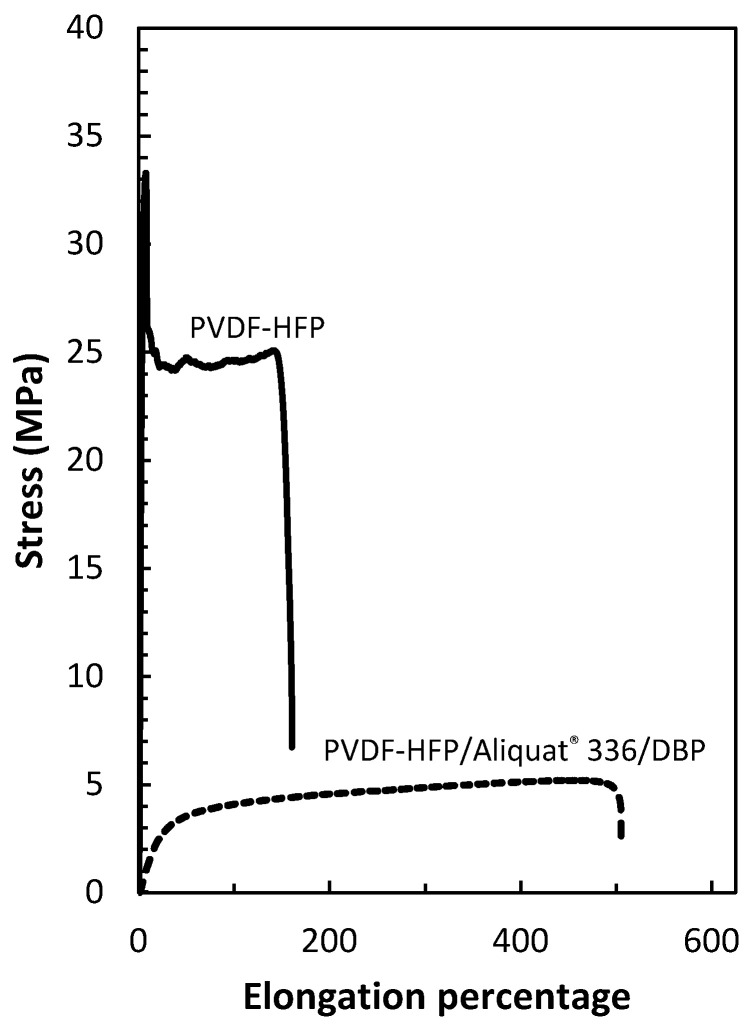
Stress–strain diagrams for the PVDF–HFP film and the optimized PIM (50 wt% PVDF-HFP, 40 wt% Aliquat^®^ 336 and 10 wt% DBP).

The stress–strain curves of the blank PVDF-HFP film and the optimized PIM (50 wt% PVDF-HFP, 40 wt% Aliquat^®^ 336 and 10 wt% DBP) (Figure 14) showed that the presence of the membrane liquid phase in the optimized PIM led to a drastic reduction in tensile stress, i.e., from 33.2 to 5.2 MPa, in the peak point. More importantly, the addition of extractant and plasticizer/modifier resulted in significant increase in flexibility, reaching a maximum at around 500% of elongation.

## 4. Conclusions

The present study showed that a PIM composed of 50 wt% PVDF-HFP, 40 wt% Aliquat^®^ 336 and 10 wt% DBP was suitable for the selective extraction of V(V) from its sulfate solutions in the presence of Al(III), Co(II), Cu(II), Fe(III), Mn(II) and Ni(II), which are often encountered in digests of spent alumina hydrodesulfurization catalysts. The co-extraction of Mo(VI) was eliminated in a two-step process where Mo(VI) was extracted first into a PIM at higher acidity. Quantitative back-extraction of V(V) was achieved in a back-extracting solution containing 6 mol L^−1^ H_2_SO_4_ and 1 *v*/*v*% H_2_O_2_. The extraction of V(V) was suggested to be based on the exchange of the Aliquat^®^ 336 chloride anions with VO_2_SO_4_^−^, while the formation of the VO(O_2_)^+^ in the back-extraction process as a result of the oxidation of VO_2_^+^ to VO(O_2_)^+^ by H_2_O_2_ was assumed to play a key role in the quantitative back-extraction of V(V).

## Figures and Tables

**Table 1:** Comparison of the extraction percentages of the successful PIMs ^a^.

PIM	PVDF-HFP (wt%)	Aliquat^®^ 336 (wt%)	DBP (wt%)	Extracted V(V) ± SD (%)
1	50	35	15	61.41 ± 0.82
2	55	35	10	61.48 ± 1.06
3	55	40	5	62.19 ± 0.49
4	50	40	10	63.80 ± 0.42

^a^ The remaining experimental conditions are outlined in Figure 2.

**Table 2:** Extraction and back-extraction percentages of V(V) from its solutions containing other metallic species ^a,b^.

Solution	Metallic Species	Extraction (%)	Back-Extraction (%)
1	V(V)	76.3 ± 0.6	76.1 ± 0.6
2	V(V) Mo(VI)	52.2 ± 2.1 98.6 ± 2.6	51.7 ± 1.5 20.0 ± 2.0
3	V(V) Al(III)	72.3 ± 0.7 2.6 ± 0.9	71.4 ± 0.4 ND
4	V(V) Co(II)	73.9 ± 1.1 0.6 ± 2.0	73.6 ± 1.3 ND
5	V(V) Cu(II)	74.4 ± 1.9 ND	74.0 ± 0.7 ND
6	V(V) Fe(III)	75.2 ± 2.1 ND	75.0 ± 1.8 ND
7	V(V) Mn(II)	76.1 ± 1.6 ND	76.0 ± 1.8 ND
8	V(V) Ni(II)	73.2 ± 2.2 ND	72.9 ± 1.3 ND
9	V(V) Mo(VI) Al(III) Co(II) Cu(II) Fe(III) Mn(II) Ni(II)	50.4 ± 1.3 99.3 ± 2.6 ND ND ND ND ND ND	49.6 ± 0.5 19.8 ± 2.1 ND ND ND ND ND ND

^a^ PIMs: 3.5 cm diameter circular segments containing 50 wt% PVDF-HFP, 40 wt% Aliquat^®^ 336 and 10 wt% DBP. Extraction: initial aqueous source solution: 50 mL with V(V) alone or with other ions (each 50 mg L^−1^) adjusted to 0.2 mol L^−1^ sulfate and pH 2.5, extraction time 24 h. Back-extraction: 50 mL 6 mol L^−1^ sulfuric acid and 1 *v*/*v*% hydrogen peroxide, back-extraction time 24 h. ^b^ ND—not detectable.

**Table 3:** Results of a two-step extraction/back-extraction procedure for the selective PIM-based separation of V(V) from a source solution containing Mo(VI), Al(III), Co(II), Cu(II), Fe(III), Mn(II) and Ni(II) (50 mg L^−1^ each) ^a,b^.

Ionic Species	pH 1.1 (Step I)		pH 2.5 (Step II)	
	Extraction (%)	Back-Extraction (%)	Extraction (%)	Back-Extraction (%)
V(V)	ND	ND	74.2 ± 1.9	73.7 ± 1.4
Mo(VI)	96.4 ± 1.2	19.6 ± 2.6	ND	ND
Al(III)	ND	ND	ND	ND
Co(II)	ND	ND	ND	ND
Cu(II)	ND	ND	ND	ND
Fe(III)	ND	ND	ND	ND
Mn(II)	ND	ND	ND	ND
Ni(II)	ND	ND	ND	ND

^a^ Experimental conditions: aqueous source solution—50 mL, back-extraction solution: 50 mL, 6 mol L^−1^ H_2_SO_4_ and 1 *v*/*v*% H_2_O_2_, PIM composition: 50 wt% PVDF-HFP, 40 wt% Aliquat^®^ 336 and 10 wt% DBP, extraction and back-extraction time 24 h. ^b^ ND—not detectable.
